# Internet-based attentional bias modification training as add-on to regular treatment in alcohol and cannabis dependent outpatients: a study protocol of a randomized control trial

**DOI:** 10.1186/s12888-017-1359-2

**Published:** 2017-05-23

**Authors:** Janika Heitmann, Madelon E. van Hemel-Ruiter, Karin M. Vermeulen, Brian D. Ostafin, Colin MacLeod, Reinout W. Wiers, Laura DeFuentes-Merillas, Martine Fledderus, Wiebren Markus, Peter J. de Jong

**Affiliations:** 1 Verslavingszorg Noord Nederland, Leonard Springerlaan 27, 9727 KB Groningen, The Netherlands; 2 Experimental and Clinical Psychology, Department of Psychology, University of Groningen, Grote Kruisstraat 2/1, 9712 TS Groningen, The Netherlands; 3 Department of Epidemiology, University Medical Center Groningen, University of Groningen, P.O. Box 30.001, 9700 RB Groningen, The Netherlands; 4 School of Psychological Science, The University of Western Australia, 35 Stirling Highway, Crawley, WA 6009 Australia; 5 Department of Psychology, University of Amsterdam, Nieuwe Achtergracht 129B, 1018 WS Amsterdam, The Netherlands; 6 Novadic-Kentron, Network for Addiction Treatment Services, Hogedwarsstraat 3, 5261 LX Vught, The Netherlands; 7 Tactus Verslavingszorg, Keulenstraat 3, 7418 ET Deventer, The Netherlands; 8 Iriszorg, Kronenburgsingel 545, 6831 GM Arnhem, The Netherlands

**Keywords:** Addiction, Attentional bias modification, eHealth

## Abstract

**Background:**

The automatic tendency to attend to and focus on substance-related cues in the environment (attentional bias), has been found to contribute to the persistence of addiction. Attentional bias modification (ABM) interventions might, therefore, contribute to treatment outcome and the reduction of relapse rates. Based on some promising research findings, we designed a study to test the clinical relevance of ABM as an add-on component of regular intervention for alcohol and cannabis patients.

**Design/Methods:**

The current protocol describes a study which will investigate the effectiveness and cost-effectiveness of a newly developed home-delivered, multi-session, internet-based ABM (iABM) intervention as an add-on to treatment as usual (TAU). TAU consists of cognitive behavioural therapy-based treatment according to the Dutch guidelines for the treatment of addiction. Participants (*N* = 213) will be outpatients from specialized addiction care institutions diagnosed with alcohol or cannabis dependency who will be randomly assigned to one of three conditions: TAU + iABM; TAU + placebo condition; TAU-only. Primary outcome measures are substance use, craving, and rates of relapse. Changes in attentional bias will be measured to investigate whether changes in primary outcome measures can be attributed to the modification of attentional bias. Indices of cost-effectiveness and secondary physical and psychological complaints (depression, anxiety, and stress) are assessed as secondary outcome measures.

**Discussion:**

This randomized control trial will be the first to investigate whether a home-delivered, multi-session iABM intervention is (cost-) effective in reducing relapse rates in alcohol and cannabis dependency as an add-on to TAU, compared with an active and a waiting list control group. If proven effective, this ABM intervention could be easily implemented as a home-delivered component of current TAU.

**Trial registration:**

Netherlands Trial Register, NTR5497, registered on 18th September 2015.

## Background

Alcohol and drug use disorders are well known for their persistent character. People diagnosed with substance use disorders are usually well aware of the undesirable consequences of their substance use and, therefore, often desire to quit. However, in spite of their motivation to stop, they frequently report an inability to voluntarily control their alcohol or drug use. This loss of control is associated with high rates of relapse, which have been found to be 40–50% one year after successful treatment, rising to 70% three years later [1, 2]. These numbers indicate that current interventions might not successfully address all relevant aspects of addiction. Therefore, (cost-) effective methods of improving existing interventions need to be developed, in order to lower rates of relapse and thereby increase patients’ quality of life.

Most current interventions in addiction care, such as cognitive behavioural therapy (CBT), focus on conscious decision-making and behavioural control, such as learning how to recognize ‘risky’ situations, and to unravel and change possible ‘wrong beliefs’ about the alcohol or drug use. This restricted focus of current interventions on conscious processes might be a possible reason for their limited success rates. Accordingly, current dual process models of addiction emphasize that next to the conscious cognitive processes also more automatic processes, such as attentional bias, play a crucial role in the development and persistence of addiction [3, 4]. *Attentional bias* in substance dependency can be defined as an automatic tendency to focus attention on substance-related cues in the environment [5, 6], such as alcohol advertisement or a used joint on the street. Attentional bias for substance-related cues has repeatedly been found in different substance use disorders, such as alcohol, tobacco, and opioids dependency [7–9]; for a critical review on the role of attentional bias in addiction see [10].

Heightened attention to substance-related cues can involve two processes. The first process is attentional engagement bias, reflecting increased direction of attention towards substance-related cues. The second process is attentional disengagement bias, reflecting increased subsequent difficulty disengaging attention from substance-related cues. In addiction, both types of attentional bias appear to operate. Patients diagnosed with substance use disorder are likely to become quickly aware of substance-related cues in their environment [11], plausibly indicating that attention is spontaneously drawn towards these cues. Furthermore, heavy users seem to focus their attention longer on substance-related information than do social users [12], which suggests that heavy users experience difficulty disengaging their attention away from substance-related cues.

Both biases in attention will increase awareness of substance-related cues, which might be especially problematic for people who would like to stop or to reduce their intake of alcohol or drugs [13]. That is, patients diagnosed with substance use disorders seem to be surrounded by relatively many temptations, which makes resisting even more challenging. Recent research supports the idea that attentional bias towards alcohol and drug cues is related to the intensity and persistence of addictive behaviours. First, attentional bias has been found to increase in strength during the course of more frequent and increased use of the addictive substance [14]. In other words, the strength of attentional bias favouring substance-related cues is related to the severity of addiction. This may reflect a self-reinforcing bias-use-bias cycle, in which increased substance use induces increased attentional bias which in turn increases substance use and so on. This may render it increasingly difficult to quit the use of addictive substances. Second, higher levels of subjective craving have been found to be related to stronger attentional bias favouring substance-related cues [15]. Such increased attention to substance-related information may increase the desire to use and, as a result, interfere with the deliberate intention limiting or stopping the intake of alcohol or drugs. Third, greater attentional bias to substance-related cues prior to treatment has been shown to be related to poorer treatment outcome [16], indicating that people who display greater attentional bias to substance-related cues benefit less from current interventions than people who exhibit little or no such attentional bias. Finally, some studies have found that strong attentional bias to substance-related cues increases the risk of relapse after successful treatment [17]. Importantly, recent studies have found that this attentional bias is largely unaffected by current CBT-based interventions [11, 18], suggesting that an intervention aimed at directly reducing attentional bias to substance-related cues might add to the effects of traditional CBT.

In line with this, a new type of interventions has been developed with the specific aim of modifying biased information processes, collectively called cognitive bias modification (CBM) interventions. The subset of CBM interventions specifically targeting attentional bias are referred to as attentional bias modification (ABM) interventions. Both in the context of experimental research and clinical trials, it has been shown that attentional bias can be effectively altered using computerized procedures [17, 19, 20]. However, in order to achieve clinically meaningful effects it is important that reduced attentional bias results in changes of substance use-related symptoms. Of course, if an ABM intervention does not modify attentional bias, then no changes in symptoms or behaviour can be expected [21, 22], and ABM interventions are unlikely to be effective when attentional bias is not present prior to the intervention [23–25]. However, if an ABM intervention does result in the reduction of pre-existing attentional bias, then it becomes plausible that there also will be corresponding reduction in symptoms that are in part caused or maintained by this attentional bias, and such changes in clinical symptoms can be evaluated. It has been observed that in recent research the successful modification of attentional bias to substance-related cues has often not been transferred into these desired behavioural changes, such as reduced alcohol or drug intake or increased time until relapse [20, 26]. Therefore, it seems important to consider the factors that may moderate whether the modification of such attentional bias delivers these clinically meaningful benefits.

One important factor may be motivation to change. In most of the experimental studies in which attentional bias to substance-related cues has been modified successfully, without corresponding change in problematic substance use behaviour, participants have been non-clinical volunteers without apparent motivation to change their substance use [19, 20, 27]. To test the capacity of ABM interventions to attenuate addiction-related symptoms in clinical cohorts, it is necessary to deliver ABM interventions to samples of treatment-seeking participants; patients who are motivated to change their problem behaviour. In line with this, recent research in an outpatients clinical sample found that adding an ABM intervention to regular treatment not only led to greater reduction in attentional bias to substance-related cues, but also to longer times until relapse, when compared with a placebo control condition [17]. Such findings are consistent with the idea that, when patients are motivated to change, the direct modification of attentional bias to substance-related cues can add to the efficacy of conventional interventions.

Another factor that may moderate the efficacy of ABM interventions is the context in which the intervention is delivered. Within the field of social anxiety research, interventions designed to reduce attentional bias to social threat cues have been shown to yield beneficial effects on social anxiety when delivered in the laboratory or clinic [28, 29], but often do not deliver such benefits when administered as home-delivered interventions [30]. Typically, in the home environment, these interventions fail to alter attention to social threat cues. A possible explanation might be that people diagnosed with social anxiety disorder experience their home environment as a safe place, and do not display attentional vigilance for threat cues or experience anxiety in their home setting. This lack of anxiety and the absence of attentional bias prior to the intervention might interfere with its efficacy [31]. In line, the theory of emotional processing suggests that fear-relevant information needs to be activated in order to change it successfully [32]. In contrast, in substance use disorders the experience of craving can be expected to be experienced most strongly in the home environment, as craving is likely to be strongest within the environment in which people typically tend to use [33]. As the laboratory or clinic are novel environments, these are not likely to induce craving and thus ABM interventions might be less effective when delivered in these types of substance-use irrelevant environments. Thus, ABM interventions for substance dependency might be even more effective when delivered at home and when the experience of craving is high. However, in apparent conflict with this, a study in which an ABM intervention was delivered via the internet failed to find convincing support for its efficacy [34]. Although participants in the ABM group showed a reduction in the consumed glasses of alcohol a day, this effect could not be attributed to the intervention, because the same effect was found in the placebo control group. Importantly, this study delivered a web-based ABM intervention in the absence of any other (motivational) intervention, and participants did not intent to stop or to reduce their alcohol use. In order to clarify the effectiveness of home-delivered ABM interventions in substance dependency more research accounting for all important factors is needed.

A potentially important limitation of previous studies, which may have constrained their therapeutic impact, concerns the simplicity of current attentional training tasks that have been employed with the aim of altering real world attentional bias. Typically, these tasks have presented only two static stimuli, one of which is related to the target category of information, such as alcohol, and participants have been required to discriminate the identity of a small “probe” that appears either in the locus of this stimulus or in the locus of the other neutral stimulus. By presenting these probes distally from the target stimulus, it is hoped that participants will come to attend away from this category of information [35]. This simple task, displaying only two static stimuli, does not challenge the attentional system, and clearly lacks the dynamic complexity of real world settings, which may limit transfer of the resulting training effects to real-life situations [36]. Hence, it would be desirable to develop more complex and dynamic ABM interventions in order to train change in attentional vigilance to substance-related information that is most likely to transfer into real-world settings, and so drive therapeutic changes in substance use-related behaviour and symptoms.

Another important potential consideration is the number of training sessions that should be included in an ABM intervention. Laboratory studies have shown that a single session of ABM intervention can modify attentional bias transiently, but studies using this approach to alter attention to substance-related cues have not found generalization of such effects to new stimuli that were not employed in the training procedure nor changes in substance use-related symptoms [19, 20, 37]. This suggests that multiple sessions of ABM intervention are likely to be necessary for the modification of attentional bias to generalize to novel stimuli, and translate into relevant changes in substance use-related symptoms. In line with this, preliminary evidence shows that the modification of attentional bias to substance-related cues through multiple sessions can result in changes of substance use-related behaviour [17]. Given the need to deliver multiple training sessions to produce clinically meaningful change in symptoms, it is necessary to ensure that participant motivation is sustained. This, it may be advisable to add motivational components to the ABM intervention, either face to face or online. Such components, which may include motivational interviewing, could be designed to increase motivation to change substance use [38] or motivation to finish the ABM intervention in particular [39]. Another way to increase motivation to remain engaged in a multi-session ABM intervention would be to make the training itself more appealing, for example via gamification of the intervention [40, 41]. Of course, while such gamification is likely to increase motivation to perform the training, it may not enhance motivation to change addictive behaviour [40]. Therefore, it will be important to sustain regular treatment, including motivational components.

Based on these considerations, the current study will investigate the effects of a gamified, home-delivered, and internet-based multi-session ABM intervention as an add-on to regular face-to-face protocolled TAU. This combined treatment may strengthen the motivation to change by sustaining treatment as usual (TAU) on the one hand, while also directly reducing attentional bias favouring substance-related cues (ABM intervention) on the other hand, leading to improved treatment outcome and decreased rates of relapse.

### Trial objective and hypothesis

The current study will investigate the effectiveness of a newly developed internet-based ABM (iABM) training as an add-on intervention to treatment as usual (TAU; consisting of protocolled CBT-based interventions), in alcohol and cannabis dependent outpatients. More specifically, the study will evaluate whether this new iABM training enhances treatment outcomes, and may be a cost-effective component that should be added to treatment in addiction care. The focus of the study is to examine the additional effects of iABM training on changes in substance use, craving, and relapse rates. Measurements of attentional bias will serve as a manipulation check for the efficacy of the current training to successfully modify attentional bias. This is important because only if the current iABM intervention is effective in modifying patients’ attentional bias it can be expected to have an impact on their substance use.

Based on this objective, we will test the hypothesis that patients who receive iABM intervention, compared to controls, will show less substance use, less craving, and lower relapse rates at post-measurement and 6 and 12 months after treatment. In addition, we will test if patients who received iABM intervention will show increased health and reduced physical and psychological complaints. Furthermore, we predict that these patients will show a reduction in health care usage 6 and 12 months after the intervention. Moreover, we predict that the effects on the individual and societal level will cause a decrease in societal costs that outweighs the additional costs of iABM intervention. Finally, we hypothesize that participants who show a stronger attentional bias and higher rates of craving before starting the ABM training will benefit most from this intervention.

## Methods and design

### Trial design

**Fig. 1:**
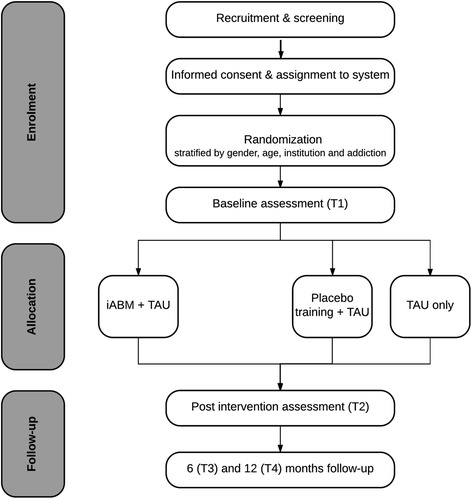
Participant flowchart

**Fig. 2:**
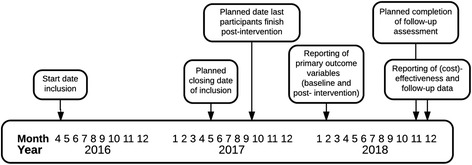
Expected timeline of completion and reporting of results

The present study is a multicentre randomized controlled two-armed, parallel-designed trial with one treatment arm (iABM intervention) and a control arm, which will be divided into a placebo condition and a TAU-only condition (see Fig. 1). The inclusion of these two control conditions enables (1) the investigation of the effect of TAU + iABM intervention, related to TAU-only, and (2) the control for a possible placebo effect, by comparing the iABM condition with the placebo condition. The design of the study enables that iABM intervention, as well as the placebo condition, can be provided parallel to treatment as usual (TAU). Recruitment will take place in four addiction care centres in the Netherlands (*Iriszorg*, *Novadic-Kentron, Tactus Verslavingszorg and Verslavingszorg Noord Nederland*). There are two treatment intensities in the Dutch mental health care system. All participants who will be included in this study will receive the lower dose treatment, in which TAU consists of 350 to 750 min of protocolled CBT-based intervention in a specialized addiction care institution, including a 30% range of possible additional interventions, such as medication. Whether therapists make use of such additional intervention components depends on the severity of addiction and possible related problems of the patients. Patients who are ascribed to this intensity of treatment are expected to live relatively independent and to report less comorbidity. The additional effects of the iABM intervention will be tested directly after the end of TAU, which is also the end of the training, and 6 and 12 months later. For an overview of the planned timeline of the study, see Fig. 2.

This study was approved by the ethical committee of the University Medical Centre of Groningen (UMCG; METc 2016/026) and is registered at the Netherlands Trial Register (NTR5497). It is partially funded by ZonMw (The Netherlands Organisation for Health Research and Development; 80–84,300–98-61,035), and co-financed by Verslavingszorg Noord Nederland. Modifications to the study protocol will be communicated with the ethical committee as well as with ZonMw. This trial protocol is written in adherence with the Standard Protocol Items:

Recommendations for Interventional Trials (SPIRIT) guidelines.

### Participants and procedure

Participants will be treatment-seeking adult patients diagnosed with alcohol use disorder (AUD) or cannabis use disorder (CUD) with an indication for TAU in addiction care as described above. Recruitment will take place at the four addiction care centres. The therapists will inform all their patients during the intake or the first session of TAU about the possibility of participating in a study. Interested patients will receive a folder, containing a leaflet from the Dutch government about scientific research in general, a patient information letter with specific information about the current study, an informed consent form with envelope, and a small card with contact information of the studies’ helpdesk. If patients give permission to their therapist, he/she will forward the patients’ contact information to a researcher, who will call them a few days later in order to screen for eligibility and to give a brief oral explanation about the study rationale and the norms of data processing. Thus, recruitment will be done by the researchers, while the therapists are responsible for providing the first information to their patients. Patients will be included if they (a) are 18 years or older, (b) have a main diagnosis of AUD or CUD, and (c) have an indication for TAU in addiction care as described above. A contra-indication for participation is (a) a problem with compulsive gaming, gambling disorder, or internet addiction as measured with a short version of the C-VAT 2.0 [42] and/or (b) the absence of a personal computer or laptop and/or no access to internet. Eligible patients who decide to participate will be asked to sign the informed consent form and to send it to the researcher. After receiving the signed informed consent form, they will be assigned to the online registration and monitoring tool of the study. The signed informed consent form needs to arrive at the researchers’ office within the period of the first three sessions of TAU. Otherwise the potential participant is excluded from the study, as early effects of TAU could distort pre-measures too much.

After assignment to the system, all participants will be invited by e-mail to complete the baseline assessment. Participants who are assigned to the TAU-only condition will thereafter receive TAU. Participants who are assigned to one of the trainings (active or placebo) will thereafter read the training instructions. Furthermore, they can watch an animated video in which the training is explained. Hereafter a short 5-min practice session of the training follows. One day after completing the baseline assessment and the practice session of the training, participants in one of the training conditions will receive an invitation for the first training session by e-mail. The following three weeks, participants will be asked to train on a daily basis. Thereafter, the number of training sessions per week will decline. Participants will train three times a week for another three weeks and finally once a week until the end of TAU. Thus, the actual number of training sessions is dependent on the duration of TAU. The lower doses treatment in the Dutch addiction care usually takes 3 to 6 months, depending on the severity and the progress of the patient. During the whole training period a researcher will monitor the process of the patients and whether they train regularly. After three training sessions are missed, patients will receive an automatic reminder by e-mail. If this does not result in the continuation of the training the researcher will contact the patient via the preferred way (phone, e-mail, text message or app) in order to ask whether there are any problems or doubts about the participation in the study.

An important part of the study is the involvement of the therapists. In order to improve adherence, the researchers will train the therapists before the inclusion of participants starts. The training will consist of the following parts (1) general background knowledge about attentional bias and ABM, (2) design and important parts of the study, and (3) the role of the therapists. During the training all therapists will receive a protocol in which all important aspects are described. In the first session of TAU after patients are assigned to the study, therapists will identify the time of the day patients’ craving is strongest and will instruct the patient to complete the ABM task at this particular time of the day. Furthermore, during each following treatment session of TAU, the therapist will ask the participant whether he/she trains on a regular basis, and will engage in motivational counselling if this is not the case.

During the last week of TAU in addiction care, participants in the training conditions will receive the last training invitation by e-mail. After completion, they will be invited for the post-assessment. Participants in the TAU-only condition will also receive the invitation for the post-assessment in their last week of TAU. Finally, 6 and 12 months after the end of TAU, all participants will receive an invitation for the follow-up measurements by e-mail. In case a participant does not respond to these invitations, the researcher will remind the patient via the preferred way (phone, e-mail, text message or app). Throughout the length of the whole project, a sounding board group will meet four times to monitor the process of the study. The group will include a researcher, a therapist, and a member of the client council of each addiction care centre, and an implementation professional.

### Interventions

#### Attentional bias modification training

In this study, the approach adopted to train patients to reduce attention to substance-related cues will be a variant of the recently developed *bouncing image training task (BITT),* based on the *follow the face task* that was originally designed by Colin MacLeod and colleagues (Notebaert L, Grafton B, Clarke PJF, Rudaisky D, Chen N, MacLeod C: Emotion in motion: a novel approach for the modification of attentional bias, submitted). This computerized task was developed to promote attentional disengagement from substance-relevant cues and attentional engagement with neutral, substance-irrelevant cues. The task requires participants to engage attention with substance-irrelevant cues while ignoring substance-relevant cues, and to disengage attention from the currently attended locus whenever substance-relevant cues appear there.

In the current BITT, 8 squares move around a computer screen (1024 × 600 pixels). Seven of these contain substance-relevant images, whereas one contains a substance-irrelevant image. Participants are instructed to attentionally follow the moving substance-irrelevant image and keeping their mouse cursor in the locus of this image. The challenging part of the task is that the images in all eight squares change at frequent unpredictable time intervals. Most of the time, the substance-irrelevant image will change into another substance-irrelevant image (e.g., from tea to water or from pen to post-it), while all the substance-relevant images change to other substance-relevant images, meaning that participants must maintain attention on and track the same image. However, on random occasions the substance-irrelevant image will change into a substance-relevant image, and one of the substance-relevant images will change to a substance-irrelevant image. At this point, participants must immediately disengage their attention from the square they were previously tracking, which now contains a substance-relevant image, and switch their attention to engage as fast as possible with the square that now contains the substance-irrelevant image. As soon as the participants locate the mouse cursor on the substance-irrelevant image, this image becomes green-filtered for 500 ms, so that participants know they are tracking the ‘right’ image. Each training session is divided into four blocks of 2.5 min.

To enhance the motivation of participants to train regularly and to make the training more appealing, some game-like features were added to the original version of the BITT. First, the training consists of 12 different levels, gradually increasing in difficulty. The construction of the levels is based on three factors: Moving speed of squares, interval of switching of images within squares, and interval of switching of images between squares. In order to adapt the difficulty of the levels to the abilities of the current population, the speed of the levels was tested in a small group of patients diagnosed with AUD and CUD in one of the addiction care centres during the test phase. In the beginning of the training phase, all participants start with level one and thereafter can unlock more challenging levels by reaching a certain amount of points. This cut-off score is 80, which equals a tracking time of 2 min per block. The high-scores of each level are stored, so that participants can challenge themselves by reaching higher scores during the next block or training session. Participants are instructed to choose a level that is challenging, but not too difficult and thereby frustrating. The number of trials depends on the level at which participants are training. Second, participants receive feedback about their performance in the form of points, calculated for each block. The more accurately participants track the substance-irrelevant image with the mouse cursor, the higher their score. While training, the participants’ accruing score is indicated by the length of a green bar shown on the screen. A mark on the bar gives them feedback about whether they already unlocked the next level. Next to the bar, they can also see the remaining time per block. There are two versions of the BITT: One contains stimuli that are relevant for patients diagnosed with AUD, whereas the other contains stimuli relevant for CUD patients.

To test the effect of the BITT on participants’ ability to engage attention with substance-irrelevant cues while ignoring substance-relevant cues, we will test the increase in tracking time of the substance-irrelevant cue over the course of treatment. To test the effect of the BITT on participants’ ability to disengage attention from the currently attended locus whenever substance-relevant cues appear there, we will examine the change in switching time over the course of treatment. To measure transfer of the improvement in BITT performance in terms of enhanced orientation towards substance-irrelevant cues and enhanced ability to disengage from substance cues, we also included a visual search task to measure attentional bias (see below).

#### Placebo condition

The placebo condition is designed to be similar to the active BITT, and the stimuli, the design/layout, the temporal parameters, and the construction of levels are all equal to the active intervention. However, in the control condition the task is not configured to reduce attention to substance-related cues.

In contrast to the active intervention, the placebo condition consists of four substance-relevant and four substance-irrelevant images moving on the screen. Participants are instructed to equally pay attention to all eight images until one of the squares turns green. They are asked to click on this green-filtered image as quickly as possible. The green filter appears in an unpredictable location at unpredictable time intervals. Throughout the task, the squares containing substance-relevant and substance-irrelevant images turn green equally often (50:50 ratio).

#### Task stimuli

**Fig. 3:**
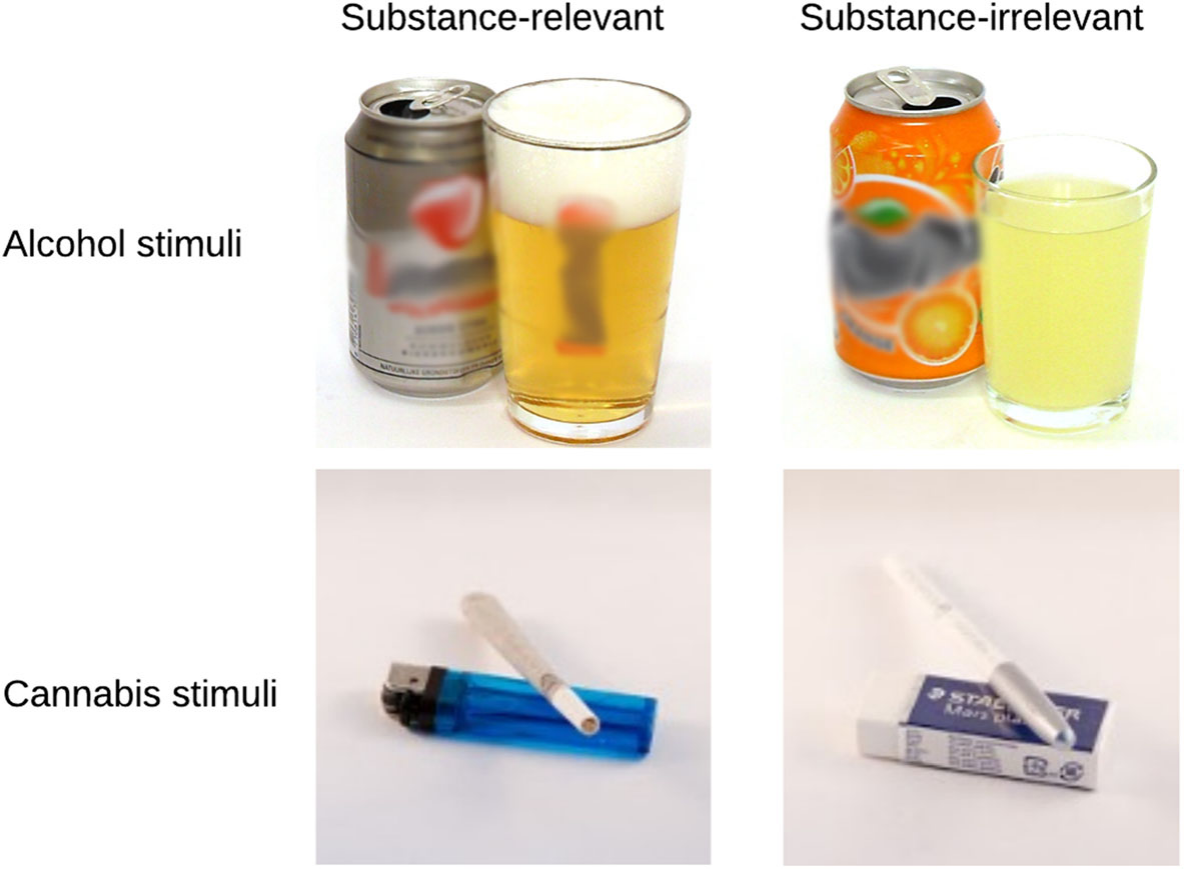
Example of training stimuli of the alcohol and cannabis version

The stimuli of the alcohol and cannabis version of the BITT and the placebo condition consist of the same two sets of 64 images (500 × 500 pixels). Whereas the first set of 64 images is used during most of the training sessions, the second set is only used for the last training session in order to measure generalization to new (untrained) stimuli. The 32 substance-relevant images of the alcohol version show different alcohol beverages, such as beer, wine, or whiskey. The substance-irrelevant category consists of 32 non-alcoholic drink images (e.g., soda, tea, or coffee). The images of both categories show a bottle, a bottle with an empty glass, or a bottle with a filled glass. The cannabis version is constructed of 32 substance-relevant images showing objects related to cannabis use (e.g., weed, joint, or rolling paper) and 32 substance-irrelevant pictures containing office products (e.g., pen, post-it, or paperclips). All used pictures were used in earlier studies [11, 43]. For each block of a training session, 8 substance-relevant and 8 substance-irrelevant images are randomly drawn from the 64 available images. For the next block, 16 other pictures will be used. Thus, throughout each training session all 64 images are presented to the participant. As images switch within an unpredictable time interval and dependent on the actual level, the number of times an image is presented within one block is not fixed. Yet, as participants reach higher levels and the speed of switching increases, each image is presented more often within one block. The background of all images is white and they are matched by colour, type of bottle/package, and size of bottle/package. Furthermore, all stimuli are passive, which means that no persons are shown in the pictures. For an example of training stimuli, see Fig. 3.

### Baseline, post measurement and follow-ups

#### Attentional bias assessment

Attentional bias to substance-related cues is measured using the *Odd One Out Search Task* approach (originally introduced by [44]], and later modified by other researchers). In this task, participants have to identify whether 20 pictures (500 × 500 pixel), presented in a 4 × 5 matrix, all belong to the same single category or whether one picture belongs to a category distinct from all the others. Dependent on the diagnosis of the participant, all pictures belong to one of the following three categories: alcoholic drinks, non-alcoholic drinks, and flowerpots or cannabis-related objects, neutral daily life devices, and flowers, respectively for AUD and CUD. Due to these three categories, there are three conditions in which no *odd one out* picture can be found in the matrix (e.g., all 20 pictures show alcoholic drinks). Accordingly, there are six possible combinations in which an *odd one out* picture is present (e.g., 19 pictures contain cannabis-related objects and one picture shows a flower). For an overview of all nine conditions per diagnoses see Table 1. The possible combinations of conditions are balanced and the order of trials is random. The task is divided into three blocks of 24 trials each, and within each block, there will be 18 trials with an *odd one out* picture and 6 trials without an *odd one out* picture. The duration of each trial is dependent on the reaction time of the participant, but last 10 s at most. Responses are given by the answer buttons on the keyboard; 1 for ‘yes, there is an *odd one out*’ and 0 for ‘no, there is no *odd one out*’. Between trials, participants are instructed to focus their attention on a red fixation cross in the middle of the screen, which is presented for 500 ms. For both versions (alcohol and cannabis), we will use a total of 90 pictures, meaning that there are 30 different pictures per category. Per trial, the 20 pictures that are presented in the matrix are randomly drawn from the available pictures. The pictures of the alcoholic and non-alcoholic drinks [43] and the pictures of cannabis-related objects and neutral daily life devices [45–48] were used in earlier studies and permission was asked. The pictures of flowerpots and flowers were taken for the purpose of the current study.

**Table 1:** Type and amount of trials in the Odd one out task, separated for AUD and CUD

Condition	Type of trials AUD	Type of trials CUD	Trials per block
1	20 alcohol images	20 cannabis-related images	2
2	20 soft drink images	20 neutral images other than flowers	2
3	20 flowerpot images	20 flower images	2
4	19 alcohol 1 soft drink	19 cannabis-related 1 neutral	3
5	19 alcohol 1 flowerpot	19 cannabis-related 1 flower	3
6	19 soft drink 1 alcohol	19 neutral 1 cannabis-related	3
7	19 flowerpot 1 alcohol	19 flower 1 cannabis-related	3
8	19 soft drink 1 flowerpot	19 neutral 1 flower	3
9	19 flowerpot 1 soft drink	19 flower 1 neutral	3

*AUD* = alcohol use disorder, *CUD* cannabis use disorder

Attentional bias scores will be calculated by subtracting the mean reaction time of trials with one neutral picture among 19 substance-relevant pictures (condition 4 and 5; see Table 1) from the mean reaction time of trials with one substance-relevant picture among 19 neutral pictures (condition 6 and 7). A positive score indicates that participants are faster in finding substance-relevant information within neutral information, than finding a neutral stimulus in an array of substance-relevant pictures, and thus reflect an attentional bias for substance-related cues.

#### Questionnaires

Participants’ general health state is assessed by means of the EuroQol-5D-3 L questionnaire [49]. Health care-related costs are evaluated with the Treatment Inventory of Costs in Psychiatric Patients questionnaire [50]. The Measurements in Addiction for Triage and Evaluation questionnaire [51] is a standard instrument in the Dutch addiction care and will be completed during the intake and at the end of the treatment through the therapists. During the 6 and 12 month follow-up measurements, the same questions will be asked online. For this study, the following parts of the MATE will be evaluated: substance use, the Obsessive-Compulsive Drinking Scale (OCDS5), and the Depression Anxiety Stress Scale (DASS).

#### Other measurements

During the baseline assessment, sociodemographic information will be collected, such as gender, relationship, level of education, and work. Furthermore, details of the patients’ own clinical history of addiction as well as of their family will be asked. Next, participants who are assigned to one of the training conditions will be asked about their expectations of the intervention. Before and after each training session, participants will rate their intensity of craving on a visual analogue scale (VAS), varying from 0 (no craving at all) to 100 (extreme craving). With this, possible direct effects of the intervention on subjective craving can be examined. Furthermore, at the end of the baseline assessment, all participants are asked to fill in a short questionnaire about their computer/laptop use in their private life. This information might be helpful when investigating for whom this iABM intervention was effective. Last, after the first week of training and during the post-assessment, participants, who are assigned to one of the training conditions, will be asked to fill in an evaluation questionnaire, in which they can give their opinion about the intervention and are asked to indicate whether the intervention helped them. Information derived from these questionnaires can be used as indications for possible next steps towards implementation. For an overview of all questionnaires and tasks per time point, see Table 2.

**Table 2:** Overview of measurement instruments per time point

Purpose	Measures	Baseline	Post-assessment	Follow-up
Attentional bias assessment	Odd one out task	X	X	X
Baseline measures	Demographics	X		
	History of addiction	X		
	Family history of addiction	X		
Primary outcome measures - clinical	MATE (substance use, craving)	X	X	X
	Clinical state (relapse)			X
Primary outcome measures - Societal/economical	TiC-P	X	X	X
	EQ-5D	X	X	X
Secondary outcome measures	MATE (depression, anxiety and stress)	X	X	X
	Computer use	X		
Others	Expectation-questionnaire	X^a^		
	Training evaluation		X^a^	

^a^only to be filled in by participants in one of the training conditions

### Primary and secondary outcome measures

The effectiveness of the iABM intervention will be examined by changes in substance use (i.e. quantity of use in the past 30 days), craving, and relapse rates. Measurements of attentional bias will serve as a manipulation check for the effects of iABM intervention.^1^ Secondary outcome measures are cost-effectiveness,^2^ and secondary physical and psychological complaints (depression, anxiety, and stress). The evaluation forms of the patients and therapists will be assessed as well.

### Sample size

To find a difference between groups of medium effect size with a power of 0.8 at an alpha of 0.05, both the treatment and the control arm need to include 64 patients, as calculated with.

G*power 3.1.5. Dropout rates in regular addiction treatment are known to be approximately 20%. Given the 6 and 12 months follow-up measurements a dropout rate of 40% was calculated. Therefore, a total amount of 213 patients will be recruited.

### Withdrawal

All participants can withdraw from the study at any time. However, they are asked whether they are willing to complete the measurements. Thus, even if participants stop the training (iABM intervention), they are still invited to participate in the post-assessment and follow-up assessments.

### Randomization

Participants who meet the inclusion criteria will be automatically assigned to one of the three conditions with the following likelihood (1) 50% TAU + iABM, (2) 25% TAU + placebo condition and (3) 25% TAU-only. Furthermore, the registration and monitoring system stratifies for gender, age group (18–30, 30–50, 50+), type of addiction, and institution. Therefore, participants are assigned to one of the three conditions to which the fewest participants of their gender, age group, and type of addiction are assigned accounting for the institution of the participants. When proven effective, participants in the control conditions (TAU + placebo condition and TAU-only) will be offered the iABM intervention after the end of the study (approx. End 2018).

### Blinding

Since all assessments take place online, thus in the absence of the researchers, the outcome data are blinded. However, one researcher will be aware of the condition of the participants in order to support them appropriately when technical or personal problems appear. It is unlikely that this will entail problems of bias, as the researcher is not involved in any measurement and the motivational part of the study lies in the responsibility of the therapists. The therapists and participants will be blinded for the two training conditions. Therefore, the iABM intervention and the placebo condition were designed such that the use of the same task stimuli would be possible, making both conditions look very similar. Furthermore, it is unlikely that participants will tell their therapists much about the content of the training, but more about whether they train regularly or whether they like or dislike the training. This will help to keep the therapists blinded. Furthermore, the therapists will be instructed not to do any ‘research’ about what might be the ‘real’ intervention. Participants will be asked to indicate their expectation about their condition in the post-assessment.

### Data analyses

#### Clinical analysis

First, changes in attentional bias, as measured with the *odd one out task*, will be examined by using a 4 (within subjects: pre, post, FU1, FU2) × 2 (between subjects: TAU + iABM versus control conditions) repeated measure ANOVA, with attentional bias as dependent variable. To assess the validity of the main hypothesis, a 4 (within subjects: pre, post, FU1, FU2) × 2 (between subjects: TAU + iABM versus control conditions) repeated measure ANOVA will be conducted to examine the effects of the iABM intervention on substance use and craving, as measured with the MATE. In order to evaluate the effects of iABM intervention on rates of relapse, a Cox regression analysis will be conducted to model the time until relapse occur.

Further, changes in secondary physical and psychological complaints (depression, anxiety and stress) will be tested by a 4 (within subjects: pre, post, FU1, FU2) × 2 (between subjects: TAU + iABM versus control conditions) repeated measure ANOVA, based on the MATE. Missing data will be handled with multiple imputation.

#### Economic evaluation

If the iABM intervention was shown to be effective, the economic evaluation will be performed from a societal perspective, which means that all relevant costs will be taken into account, regardless of who pays for them. Health-care costs, such as contacts with health care professionals, and costs of lost productivity will be calculated by multiplying the volumes of health-care with standard unit prices derived from the Dutch Manual for cost research [52].

The time horizon of the economic evaluation will be 12 months and will compare the iABM intervention to TAU-only. Cost-effectiveness and cost utility will be assessed by relating the incremental costs of the two treatments to the incremental outcomes. The primary outcome measure in the cost effectiveness analysis will be substance use and relapse rates, as measured with the MATE and additional questions regarding relapse. In addition, a cost utility analysis will be performed with quality-adjusted life years (QALYs) as primary outcome, based on the EQ-5D-3 L defined utilities. No discounting of costs and effects will be conducted, since the time horizon does not exceed 1 year.

Uncertainty surrounding the cost-effectiveness and cost utility ratios will be assessed using bootstrap analysis. In addition, cost-effectiveness acceptability curves will be used to inform decision-makers on the probability that iABM intervention is cost effective.

### Data management and safety monitoring

The proceedings of data security and storage are established in a data management plan, which was written according to the guidelines of the funding agent ZonMw. For detailed information about this plan please contact the first author. All serious adverse events will be reported to the ethical committee as well as to the funder of the study and will be discussed in case action is necessary.

## Discussion

Relapse rates in alcohol and drug use disorders remain high even after conventional treatment has been initially successful. This emphasizes the need for more (cost-) effective therapies in addiction care. In this trial protocol, we describe the design of a randomized control trial to investigate the effectiveness of an internet-based multi-session ABM training as an add-on intervention to regular face-to-face treatment (TAU) in alcohol and cannabis dependent outpatients. To the best of our knowledge, this study will be the first RCT to test the effects of an internet-based attentional bias modification intervention, integrated with a complete cognitive behavioural therapy-based treatment, on treatment outcome and relapse rates in alcohol and cannabis dependency.

One strength of this study is the integration of a new developed multi-session iABM intervention with face-to-face TAU, given by a trained therapist. On the presumption that addiction is maintained by conscious as well as automatic processes, the combination of CBT-based intervention with its focus on strengthening decision-making processes and conscious reasoning and iABM intervention with its focus on modifying relatively automatic processes involved in addiction might well increase treatment outcome and reduce rates of relapse. We will embed the iABM intervention into TAU by asking the therapists to involve the iABM training in the regular treatment sessions. Therapists will motivate the patients to train regularly and according patients will experience that both interventions are connected and constitute one treatment program. A second strength of this study is that the iABM is a home-delivered internet-based intervention, thus allowing patients to train in the same environment where they tend to experience high levels of craving. As we have argued, the home of the patients diagnosed with substance use disorder might be the best context to deliver these trainings. Home delivery will give the patients more freedom and enables them to train when the experience of craving is strongest. Furthermore, home-delivered interventions are generally less expensive than those that require delivery in the clinic. This makes iABM interventions attractive, as they would be relatively inexpensive to implement, if proven effective. A third strength of the study is that it will investigate relapse rates and long-term effects with two follow-up assessments, at 6 and 12 months after the end of TAU and iABM intervention. Gladwin, Wiers and Wiers [53] have argued that follow-up measures may be essential to determine whether treatment innovations enhance outcomes in addictions, given that high relapse rates represents the major limitation of existing interventions. Because the effects of ABM interventions might be especially relevant in situations of stress or other negative circumstances in daily life, its benefits may become evident only across the month that follow treatment. A final strength of the study is that by gamifying the iABM intervention, we hope to improve upon prior approaches to ABM interventions. This more dynamic training mirrors the reality of engaging and disengaging of attention to a greater degree than previously used ABM intervention for substance use disorders. In addition, by adding game-like features to make the training more appealing, we expect that patients will be more inclined to train on a regular basis.

To sum up, this study will contribute to the knowledge concerning the effectiveness of adding a novel ABM intervention to TAU in substance use disorders, by determining whether this serves to enhances positive treatment effects and reduces rates of relapse.

## Abbreviations

ABM: Attentional bias modification
AUD: Alcohol use disorder
BITT: Bouncing image training task
CUD: Cannabis use disorder
IABM: Internet-based attentional bias modification
RCT: Randomized control trial
TAU: Treatment as usual

## Footnotes

1 In the proposal for the ethical committee attentional bias was listed as secondary outcome measure. However, based on current knowledge attentional bias rather serves as a manipulation check for the primary outcome measures (clinical effects).

2 The cost-effectiveness analysis was listed as a primary outcome measure in the proposal for the ethical committee. However, this analysis only reveals insightful results when the treatment will be found to be effective. Therefore, we here consider cost-effectiveness as a secondary outcome measure.
